# Determinants of Antibody Response to SARS-CoV-2 Vaccines in Liver Transplant Recipients: The Role of Immunosuppression Reduction

**DOI:** 10.3390/vaccines10111827

**Published:** 2022-10-29

**Authors:** Chih-Hsien Cheng, Hao-Chien Hung, Jin-Chiao Lee, Po-Wei Huang, Po-Wen Gu, Yin Lai, Yu-Chao Wang, Tsung-Han Wu, Chen-Fang Lee, Ting-Jung Wu, Hong-Shiue Chou, Kun-Ming Chan, Chung-Guei Huang, Wei-Chen Lee

**Affiliations:** 1 Department of General Surgery, Division of Liver and Transplantation Surgery, Chang Gung Memorial Hospital, Chang Gung University College of Medicine, Taoyuan 333, Taiwan; 2 Department of Laboratory Medicine, Chang-Gung Memorial Hospital, Taoyuan 333, Taiwan; 3 Department of Medical Biotechnology and Laboratory Science, Chang-Gung University College of Medicine, Taoyuan 333, Taiwan

**Keywords:** COVID-19, vaccine, liver transplantation, immunosuppression adjustment

## Abstract

Liver transplant recipients on chronic immunosuppression show an attenuated antibody response after SARS-CoV-2 vaccination. Adjusting immunosuppressants during vaccination remains debated. We enrolled 380 liver transplant recipients receiving 2 doses of a protein subunit, mRNA, or a vector vaccine. The patients were informed to temporarily suspend immunosuppression for 2 weeks for both vaccination doses. We measured anti-live-SARS-CoV-2 spike neutralizing antibody levels at 1–2 months after the second vaccination; 83.9% of patients had humoral responses (SARS-CoV-2 NT_50_ ≥ 9.62 IU/mL) to 2 doses of vaccines. The mRNA (86.7%) and protein subunit vaccines (85%) yielded higher response rates than the vector vaccines (40.9%). Immunosuppression suspension during the two vaccinations yielded a higher response rate (91.5% vs. 57.7%). Only eight patients (2.1%) experienced transaminase level elevation of thrice the normal value (>110 IU/L) after the second vaccination. Most recovered spontaneously after resuming immunosuppression. Multivariate analysis revealed ABO incompatibility, white blood cell count <4000, lymphocyte count <20%, tacrolimus trough level >6.5 ng/mL, and no immunosuppression adjustment as independent risk factors to nonresponse. The mRNA and protein subunit vaccines yielded a higher response rate. Immunosuppression suspension for 2 weeks enhanced the antibody response. ABO incompatibility, leukopenia, lymphopenia, a high tacrolimus trough level, and no immunosuppression adjustment are associated with nonresponse.

## 1. Introduction

Clinical trials have reported an efficacy of 74–95% for currently approved SARS-CoV-2 vaccines [1,2,3]. In Taiwan, four vaccines are currently available: ChAdOx1 nCoV-19 vector vaccine (AstraZeneca, Cambridge, UK), BNT162b2 (BioNTech-Pfizer, Mainz, Germany/New York, NY, USA) and mRNA-1273 (Moderna, Cambridge, USA) messenger RNA (mRNA) vaccines, and MVC-COV1901 (Medigen Vaccine Biologics Corporation, Taipei, Taiwan) protein subunit vaccine.

The severity and mortality of SARS-CoV-2 infection is higher among transplant recipients on chronic immunosuppression [4,5]. Although SARS-CoV-2 vaccination is generally recommended [6], the efficacy of these vaccines among immunocompromised patients is unknown given their exclusion from all vaccine trials. Studies among transplant recipients have demonstrated a weaker humoral response [7,8,9,10]; however, the efficacy of SARS-CoV-2 vaccines in patients undergoing liver transplantation (LT), particularly those receiving steroids, antimetabolites, or desensitization treatment for ABO incompatible (ABOi) living donor liver transplantation (LDLT), remains unclear.

The modulation of immunosuppression regimens at time of vaccination has been recommended for patients being treated for autoimmune diseases [11], however, this strategy may not be appropriate in transplant recipients at risk of rejection. Several studies suggest that overimmunosuppression and the use of antimetabolites are risk factors for vaccine nonresponse [12,13]. In renal transplant recipients, stopping mycophenolate mofetil/mycophenolic acid (MMF) has revealed controversial results [14,15]. We therefore aimed to assess the antibody response after vaccination with different SARS-CoV-2 vaccines in a single-center cohort of patients receiving LT and we specifically analyzed the effect of temporarily holding MMF for 2 weeks from the date of vaccination.

## 2. Materials and Methods

### 2.1. Study Design and Participants

The study included patients who underwent LT and received 2 doses of SARS-CoV-2 vaccines between June and November 2021. Patients were divided into 3 groups: the BNT162b2, or mRNA-1273 + BNT162b2, or mRNA-1273 (MRNA group); the ChAdOx1 nCoV-19 + ChAdOx1 nCoV-19 (AZ group); and the MVC-COV1901 + MVC-COV1901 (KT group). Only 1 patient received the ChAdOx1 nCoV-19 + mRNA-1273 vaccination and was excluded from the study.

All patients had stable liver function and no prior COVID-19 infection at vaccinations. Antibody titers were measured approximately 1–2 months after the second vaccination.

### 2.2. Serological Testing

We isolated the plasma from the obtained blood samples within 4 h of collection. Anti-live-SARS-CoV-2 spike neutralizing antibody levels were measured as described previously [16]. We conducted the live viral culture procedures in a biosafety level 3 facility regulated by the Taiwan Center for Disease Control. Briefly, Vero E6 cells were seeded in 96-well plates and incubated overnight. Tested sera were diluted in modified Eagle’s medium (Thermo Fisher Scientific, Waltham, MA, USA) at an initial dilution factor of 20, and then further 2-fold serial dilutions were performed to a final dilution of 1:5120. SARS-CoV-2 viruses at 100 TCID50/50 μL (Wuhan wildtype, hCoV-19/Taiwan/CGMH-CGU-01/2020, GenBank accession MT192759) were mixed with sera in an equal volume and incubated at 37 °C for 1 h before adding to Vero E6 cells. The mixture was incubated at 37 °C for 5 days and then fixed using 4% formalin for 1 h and stained with 0.1% crystal violet for visualization.

A quantitative assay of the SARS-CoV-2 50% neutralization titer (NT50) was determined based on the median tissue culture infectious dose (the dilution of a virus required to infect 50% of given cell culture) and converted into an international unit (IU/mL, WHO Standardized) [17]. NT50 < 9.62 IU/mL and ≥9.62 IU/mL was considered a negative and a positive humoral response, respectively.

### 2.3. Immunosuppression Adjustment

The immunosuppression regimen consisted of tacrolimus (Prograf^®^ or Advagraf^®^, Astellas Pharma, Northbrook, IL, USA) with or without mycophenolate mofetil (Cellcept^®^, Roche, Basel, Switzerland), mycophenolate sodium (Myfortic^®^, Novartis, Basel, Switzerland), or everolimus (Certican^®^, EV, Novartis, Basel, Switzerland). Some patients had received a triple immunosuppression regimen including prednisolone, tacrolimus, and MMF or EV.

The immunosuppression regimen was not adjusted if it consisted of tacrolimus alone. In some patients, transient immunosuppression adjustment was performed if tacrolimus was combined with MMF/EV. The patients were asked to pause MMF or EV for 2 weeks from the date of vaccination.

ABOi LDLTs underwent a desensitization protocol that consisted of rituximab and bortezomib in urgent acute liver failure cases. In the routine protocol, if the IgG and IgM antibody titers were ≤1:64, the patient underwent ABOi LDLT directly [18,19]. Rituximab (375 mg/m^2^) was administered after surgery on postoperative day (POD) 1. If IgG and IgM anti-ABO titers were >1:64, rituximab (375 mg/m^2^) was administered intravenously 3 weeks before LT. If the pre-transplant isoagglutinin titers remained at >1:64, plasmapheresis or plasma exchange was performed and repeated as needed to decrease the antibodies to ≤1:64 at surgery. Additional rituximab (187.5 mg/m^2^) was administered on POD 1. In the urgent protocol for acute liver failure recipients, bortezomib (3.5 mg) was administered, and if the anti-blood type isoagglutinin titers were ≤1:64, LT was performed immediately. If the titers were >1:64, plasma exchange was arranged. Rituximab (375 mg/m^2^) was administered on POD 1 [20].

### 2.4. Clinical Follow-Up

All patients were regularly followed up at the outpatient clinic every 1–2 months. Liver function, renal function, and tacrolimus trough levels were measured at every visit.

### 2.5. Safety

Participants were asked to report the adverse effects after each vaccination. Of the recorded adverse events, the systemic ones included dizziness, fever, chills, headache, fatigue, myalgia, dyspnea, and diarrhea; the local ones included pain and pruritus.

### 2.6. Statistical Analysis

We collected data including age, sex, timing and type of vaccination, underlying diseases, laboratory tests, and immunosuppression therapy before each vaccination.

Continuous data are expressed as medians and interquartile ranges. Differences in continuous variables were assessed using the Mann–Whitney *U* or Kruskal–Wallis test. Categorical variables were expressed as proportions and analyzed using the chi-square or Fisher exact test. We used a binary logistic regression model to identify independent factors predicting the serologic response after vaccination. Variables with *p* < 0.05 were entered in the multivariate analysis. Statistical analysis was performed with SPSS 26 statistical software (IBM-SPSS Statistics; IBM Corporation, Chicago, IL, USA), and GraphPad Prism 9.0 software (GraphPad, San Diego, CA, USA) was used for illustration. A *p* < 0.05 was considered statistically significant.

No donor livers were procured from executed prisoners. The institutional review board of Chang Gung Memorial Hospital (IRB No. 202102089B0) approved the study. All patients provided written informed consent.

## 3. Results

### 3.1. Patient Characteristics

**Figure 1:**
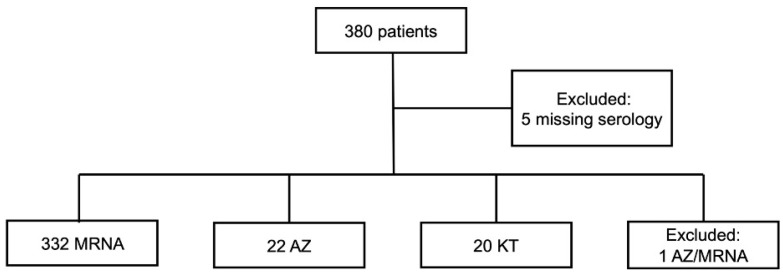
Flow chart of the study.

We included 380 patients undergoing LT (median age, 62 years; IQR, 55–67.2 years; men, 287 [76.7%]; women, 87 [23.3%]). Six patients were excluded (5 missing serology data and 1 receiving ChAdOx1 nCoV-19 + mRNA-1273 vaccination as no other included patient received the same vaccine regimen). The MRNA group was the largest by patient count (*n* = 332), followed by AZ (*n* = 22) and KT (*n* = 20; Figure 1). The most frequent indications for LT were viral hepatitis B-induced liver cirrhosis (*n* = 221, 59.1%), hepatocellular carcinoma (*n* = 153, 40.9%), and alcoholic liver cirrhosis (*n* = 73, 19.5%; Table 1). Most patients received LDLTs (*n* = 288, 77%), and 12 patients (3.2%) underwent ABO incompatible (ABOi) LTs. The median time between LT and the first vaccination was 73 months (IQR, 39–112.5 months).

### 3.2. Immunosuppression Regimens

At vaccination, 84 patients (22.5%) were receiving calcineurin inhibitor (CNI)-based monotherapy (mostly tacrolimus, 99.4%) and received no immunosuppression adjustment during the vaccinations; an additional 32 patients (8.6%) also had no immunosuppression adjustment. Of the six patients receiving triple immunosuppression (tacrolimus, MMF, and steroids), two stopped MMF during the second vaccination. Of the patients who had immunosuppression adjustments, 31 patients (8.3%) stopped MMF/EV at the second vaccination, and 227 patients (60.7%) temporarily suspended MMF/EV during both vaccinations.

### 3.3. Humoral Responses

Overall, 314 of 374 patients (83.9%) had humoral responses (SARS-CoV-2 NT_50_ ≥ 9.62 IU/mL) to both doses of vaccines (Table 1). Among the nonresponsive patients, the proportions of those with alcoholic liver cirrhosis, ABOi, leukopenia, lymphopenia, hemodialysis or immunosuppression adjustment were greater, as were those with high tacrolimus levels. The age, sex, LT-to-vaccination interval, interval between the two vaccinations, history of cancer and renal function (assessed using EGFR) did not differ between the two groups.

On univariate analysis, ABOi, white blood cell (WBC) count <4000, lymphocyte count <20%, neutrophil lymphocyte ratio <2.25, hemodialysis, tacrolimus trough level >6.5 ng/mL, no immunosuppression adjustment or only 1 adjustment predicted a failure to develop humoral response to vaccination (Table 2). On multivariate analysis, ABOi (odds ratio [OR], 6.053; *p* = 0.010), WBC count <4000 (OR, 2.841; *p* = 0.006), lymphocyte count <20% (OR, 2.648; *p* = 0.021), tacrolimus trough level >6.5 ng/mL (OR, 2.182; *p* = 0.035), and no immunosuppression adjustment (OR, 5.026; *p* = 0.002) were independent risk factors for nonresponse to vaccination.

### 3.4. Humoral Response by Type of Vaccination

**Figure 2:**
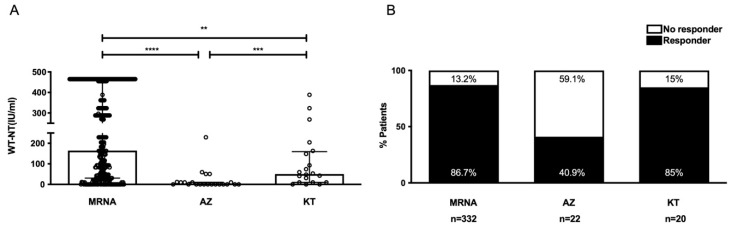
Comparison of the humoral responses (SARS-CoV-2 NT_50_ antibodies) of the LT recipients after 2 vaccinations with the mRNA, vector and protein subunit vaccines. (**A**) SARS-CoV-2 NT_50_ antibodies. Data are expressed as median and interquartile range. ** *p* < 0.01, *** *p* < 0.001, **** *p* < 0.0001 (Mann–Whitney U test). (**B**) Percentage of responders vs. nonresponders. Abbreviations: MRNA, BNT162b2 or mRNA-1273 + BNT162b2 or mRNA-1273 vaccines; AZ, ChAdOx1 nCoV-19 + ChAdOx1 nCoV-19 vaccines; KT, MVC-COV1901 + MVC-COV1901 vaccines.

The mRNA vaccines yielded a significantly higher median anti-SARS-CoV-2 antibody titer than vector or protein subunit vaccines (Figure 2). Both the MRNA and the KT groups showed higher positive response rates (86.7% and 85%, respectively) than the AZ group (40.9%). Patient characteristics differed across the three groups (Table 3). The MRNA group had a greater proportion of older patients. The AZ and KT groups had higher proportions of patients with alcoholic liver cirrhosis. Patients with ABOi were more common in the AZ group. The proportion of patients with a shorter vaccination interval was greater in the KT group. The MRNA and KT groups had a greater proportion of patients with immunosuppression adjustment.

### 3.5. Effect of Immunosuppression Adjustment

**Figure 3:**
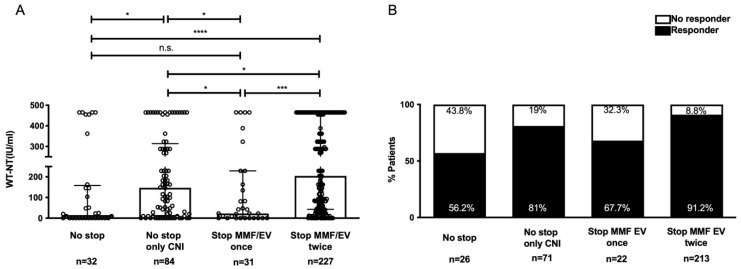
Comparison of the humoral responses (SARS-CoV-2 NT_50_ antibodies) and the proportion of responders (SARS-CoV-2 NT_50_ ≥ 9.62 IU/mL) according to immunosuppression adjustment. (**A**) SARS-CoV-2 NT_50_ antibodies. Data are expressed as median and interquartile range. n.s., not significant, * *p* < 0.05, *** *p* < 0.001, **** *p* < 0.0001 (Mann–Whitney U-test). (**B**) Percentage of responders vs. nonresponders. Abbreviations: CNI, calcineurin inhibitors; MMF, mycophenolate mofetil; EV, everolimus.

Temporary suspension of MMF/EV during the two vaccinations yielded the highest serologic response (91.5% vs. 57.7% in the group without modification; Figure 3). Of note, the antibody titers and response rates remained high (83.1%) among patients with CNI monotherapy, even without immunosuppression suspension. We observed no significant differences between MMF or EV (Supplementary Figure S1). Regarding graft safety after transient immunosuppression suspension during vaccination, serum aminotransferase levels did not differ significantly (Supplementary Figure S2). Only eight patients had a 3X elevation in transaminase levels than the normal (>110 IU/L) during follow-up after the second vaccination. Six patients recovered spontaneously after resuming immunosuppression: one patient in the no-adjustment group, three patients in the CNI monotherapy group, and two patients in the two rounds of immunosuppression adjustment group. One patient in the two rounds of immunosuppression adjustment group experienced a simultaneous biliary tract infection, which resolved with antibiotic treatment. Only one patient on the temporary 2-week MMF/EV suspension experienced acute rejection, but recovered uneventfully after pulse steroid therapy.

### 3.6. ABOi Patients

In our series, 12 patients underwent ABOi LDLT. One patient underwent deceased donor LT but was included in this group because of initial preparation for ABOi LDLT, including rituximab/bortezomib for B cell and plasma cell depletion. The median time from transplantation to vaccination in ABOi patients was 40 months (IQR, 24–81.5 months). Total bilirubin and tacrolimus trough levels were significantly higher among ABOi patients (Table 4).

**Figure 4:**
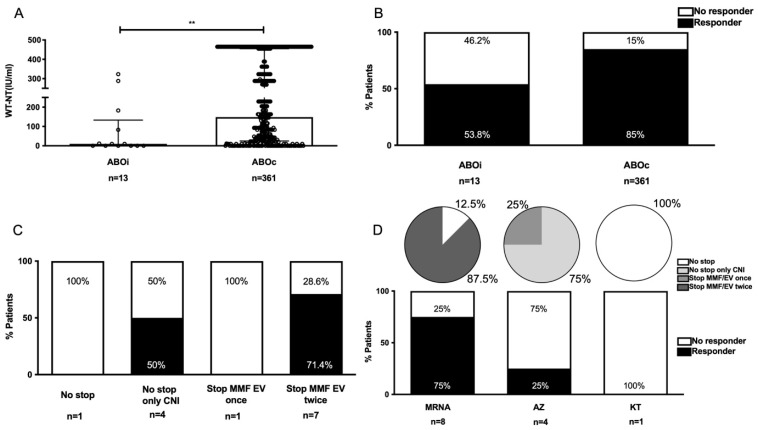
Comparison of the humoral responses (SARS-CoV-2 NT_50_ antibodies) and the proportion of responders (SARS-CoV-2 NT_50_ ≥ 9.62 IU/mL) in ABOi patients. (**A**) SARS-CoV-2 NT_50_ antibodies. Data are expressed as median and interquartile range. ** *p* < 0.01 (Mann–Whitney U test). (**B**) Percentage of responders vs. nonresponders. (**C**) Percentage of responders vs. nonresponders according to immunosuppression adjustment. (**D**) Percentage of responders vs. nonresponders according to type of vaccine and immunosuppression adjustment in each vaccination group. Abbreviations: ABOi, ABO incompatibility; ABOc, ABO compatibility; CNI, calcineurin inhibitors; MMF, mycophenolate mofetil; EV, everolimus.

Data on the percentage of CD19+ cells in the total peripheral blood lymphocyte population (%CD19) were available for only three patients and ranged between 6.7% and 14.5%. In general, ABOi patients had an impaired serological response. The median antibody titers were significantly lower, and the overall response rates were 53.8% (vs. 85% in compatible patients; Figure 4). Among patients who developed a humoral response, patients with CNI monotherapy without immunosuppression adjustment showed a 50% response rate, whereas those with two rounds of MMF/EV suspension had a response rate of 71.4%. Regarding the type of vaccines, most patients received mRNA vaccines (61.5%), and the positive immune response rate in this group was 75%.

### 3.7. Vaccination Safety

No patient who received 2 doses of any vaccine experienced any serious adverse events (Supplementary Table S1).

## 4. Discussion

Studies have shown an attenuated immune response after SARS-CoV-2 vaccination among solid organ transplant recipients [8,21,22]. However, vaccination in this population is still recommended. Understanding the factors affecting an immune response after vaccination will provide further insights for clinical practice during booster vaccinations. In this regard, this study prospectively recruited patients without prior infection undergoing LT and evaluated mRNA, vector-primed, and protein subunit SARS-CoV-2 vaccines.

The mRNA and protein subunit vaccines yielded a superior neutralizing antibody response, with response rates >85%. After 2 doses of vector vaccines, only 40.9% showed a positive response. This finding is consistent with that of previous studies showing only a 34% response rate with pure vector vaccines [21], which increased to 70% after a heterologous regimen with booster mRNA vaccines [22]. Inactivated and protein subunit vaccines remain understudied. One study reported a response rate of 17% after administering an inactivated vaccine [23]. Our study included 20 patients receiving 2 doses of a protein subunit vaccine with similar response rates to mRNA vaccines.

Similar to some other studies, our study identified ABOi leukopenia, lymphopenia, a high tacrolimus trough level, and no immunosuppression adjustment as risk factors for a failure to develop humoral response. Other factors identified were advanced age, vaccination during the first year after transplantation, hypogammaglobulinemia, a lower estimated glomerular filtration rate, the use of high-dose steroids before vaccination, and maintenance with a triple immunosuppressive regimen [8,22,24]. Therapeutic approaches that may increase the immune response to vaccination include heterologous vaccination [22], supplementation with micronutrients [25], vitamin D [26], or immunoregulatory molecules like IL-31/IL-33 [27]. However, in the short term, the most important modifiable risk factor is the immunosuppression adjustment during vaccination. In our study, we asked some patients to stop MMF or EV for 2 weeks during the two vaccinations. The response rate among patients with tacrolimus monotherapy was 81%, whereas it was 91.2% among those who suspended MMF/EV twice; this is consistent with previous reports showing decreased humoral responses with antimetabolite treatment [12,24]. MMF or mycophenolic acid therapy inhibits B-cell proliferation and plasma cell formation, which explains the reduced antibody production [28]. With EV, however, the results have been inconsistent, with studies reporting increased [29,30], attenuated [31], and unaffected [15] humoral responses after vaccination. In our series, we found no differences between suspending MMF or EV or between suspending immunosuppression once or twice during both vaccinations. Furthermore, as the liver is an immunologically tolerant organ after transplantation, the temporary suspension of MMF/EV was safe, with only 8 of 213 patients showing abnormal liver function. Most patients recovered after resuming the immunosuppression regimen after vaccination, and only one patient was admitted for steroid recycle treatment.

Regele et al. stopped mycophenolic acid or azathioprine in 18 renal transplant patients 1 week before until 1 week after the 4th/5th dose of the BNT162b2 vaccine. Although renal function remained stable and there were no new donor-specific antibodies, there were no differences in the humoral response rate vs. control or the absolute antibody levels [15]. In another cohort of 29 renal transplant patients who had not mounted a humoral response to three previous vaccine doses, Schrezenmeier et al. reported that a temporary antimetabolite treatment hold for 5 weeks increased neutralizing antibodies, vaccine-specific B cells and the ex vivo activation of spike-specific T cells. Seroconversion was already observed on day 7 in 34.4% of patients without antimetabolite treatment, as compared with only 12% of patients in the control group [14]. Data on liver transplantations are scarce, but in general, there is no consensus on immunosuppression modulation at time of vaccination in transplant patients. The effectiveness of MMF or azathioprine dose reduction before and after receiving SARS-CoV-2 vaccination is being evaluated in prospective randomized trials [32,33], which may provide further evidence to guide clinical practice.

Patients undergoing ABOi LT showed a generally inferior immunogenicity. B cell and plasma cell depletion is the primary aim of the desensitization protocols for ABOi cases, with expected recovery after 9–12 months of treatment [34]. Of interest, the only patient who underwent LT within 12 months before vaccination showed a positive immune response, highlighting the predominant effect of the increased immunosuppression and higher tacrolimus trough levels in these patients. A previous study also reported that tacrolimus trough levels in patients with a negative response was 6.6 ± 2.2 ng/mL compared with 5.4 ± 2.0 ng/mL in patients with a positive antibody response [35]. When we compared the levels of neutralizing antibodies among patients who had temporarily suspended MMF/EV, antibody levels were significantly lower when the tacrolimus trough level was >6.5 ng/mL. Therefore, when the effects of B cell depletion, MMF, or EV are minimized, adjusting the CNI trough levels is critical to achieving a humoral response.

This study has several limitations. First, we did not include a control group. However, substantial data from large clinical trials are available regarding the efficacy of vaccines in healthy individuals. Second, we did not compare the humoral responses before and during vaccinations. Although the prevalence of SARS-CoV-2 infection during the study period was very low in Taiwan, we may have missed patients with asymptomatic SARS-CoV-2 infection that likely affected the serological response. Third, we did not evaluate cellular immune responses, which is an important determinant of a durable immune response after vaccination. Fourth, we did not evaluate the longitudinal immune response, after 3–5 booster doses, or the rate of SARS-CoV-2 infection post vaccination. Sterilizing immunity is the ideal scenario, but we were unable to make any conclusions regarding the antibody response efficacy against infection or severe disease. Finally, sample sizes were very small in some groups (KT and ABOi LTs), which limited the statistical power for subgroup analysis.

In conclusion, mRNA and protein subunit vaccines yield a higher rate of neutralizing antibodies, particularly when the immunosuppression therapy is adjusted and MMF or EV is temporarily suspended. Factors associated with vaccine nonresponse included ABOi, leukopenia, lymphopenia, a high tacrolimus trough level, and no immunosuppression adjustment. These factors may provide a platform for future strategies to enhance immune responses in additional booster doses. As a proof of concept, our results highlight the effectiveness and safety of antimetabolite suspension or immunosuppression minimization during SARS-CoV-2 vaccinations.

## Figures and Tables

**Table 1:** Patient characteristics according to humoral response after vaccination.

Parameters	All Recipients (*n* = 374)	Responders (*n* = 314)	Nonresponders (*n* = 60)	*p*-Value
Age (years)	62 (55–67.2)	62 (55–67)	63 (52.2–68)	0.993
Gender				0.310
Male	287 (76.7)	244 (77.7)	43 (71.7)	
Female	87 (23.3)	70 (22.3)	17 (28.3)	
Etiology				
HCC	153 (40.9)	132 (42)	21 (35)	0.310
Viral hepatitis	221 (59.1)	190 (60.5)	31 (51.7)	0.335
HBV	58 (15.5)	45 (14.3)	13 (21.7)	0.202
HCV	13 (3.5)	12 (3.8)	1 (1.7)	0.150
HBV + HCV	73 (19.5)	55 (17.5)	18 (30)	0.404
Alcohol	18 (4.8)	16 (5.1)	2 (3.3)	0.025
Autoimmune				0.749
Type of LT				0.461
DDLT	86 (23)	70 (22.3)	16 (26.7)	
LDLT	288 (77)	244 (77.7)	44 (73.3)	
Time from LT to vaccination (months)	73 (39–112.5)	72.5 (40.7–119.2)	75.5(38–91.7)	0.334
Interval between Vaccinations (days)	84 (44–97)	84 (44–97.2)	84 (43–95.5)	0.455
ABOi	13 (3.5)	7 (2.2)	6 (10)	0.009
EGFR (mL/min/1.73 m^2^)	35.6 (20–66.2)	35.2 (20.3–66.4)	39 (16.2–63.7)	0.631
Total bilirubin (mg/dL)	0.7 (0.5–1.1)	0.7 (0.5–1)	0.7 (0.5–1.2)	0.540
AST (U/L)	20 (16–27)	20 (16–26.2)	23 (17–37.7)	0.050
ALT (U/L)	21 (14–35)	20 (14–33)	22.5 (14–49)	0.164
WBC (/uL)	5700 (4600–6900)	5900 (4700–7000)	5100 (3800–6475)	0.004
Lymphocyte (%)	28.1 (22.3–34.5)	28.5 (22.8–34.7)	25.4 (17.5–32.3)	0.030
Tacrolimus trough level (ng/mL)	4.7 (3.5–6.4)	4.6 (3.5–6.4)	5.5 (3.2–8.8)	0.208
Age > 65 (years)	135 (36.1)	109 (34.7)	26 (43.3)	0.203
Time from LT to vaccination < 12 (months)	20 (5.3)	17 (5.4)	3 (5)	1.000
History of cancer	162 (43.3)	138 (44.1))	24 (40)	0.559
WBC < 4000 (/uL)	55 (14.7)	38 (12.1)	17 (28.3)	0.001
Lymphocytes < 20 (%)	68 (18.2)	49 (15.6)	19 (31.7)	0.003
NLR < 2.25	190 (50.8)	167 (53.2)	23 (38.3)	0.035
EGFR <30 (mL/min/1.73 m^2^)	167 (44.7)	140 (44.6)	27 (45)	0.953
Hemodialysis	26 (7)	18 (5.7)	8 (13.3)	0.049
Tacrolimus trough level >6.5 (ng/mL)	87 (23.3)	66 (21)	39 (65)	0.019
No immunosuppressants stopped (CNI + MMF/EV)	32 (8.6)	18 (5.7)	14 (23.3)	0.000
No immunosuppressant stopped (CNI only)	84 (22.5)	68 (21.7)	16 (26.7)	0.394
Stopped once (MMF/EV)	31 (8.3)	21 (6.7)	10 (16.7)	0.018
Stopped twice (MMF/EV)	227 (60.7)	207 (65.9)	20 (33.3)	0.000
Triple immunotherapy	6 (1.6)	3 (1)	3 (5)	0.055

Variables are expressed as median (interquartile range) or as number (*n*) and percent (%). Abbreviations: HCC, hepatocellular carcinoma; HBV, hepatitis B virus; HCV, hepatitis C virus; LT, liver transplantation; DDLT, deceased donor liver transplantation; LDLT, living donor liver transplantation; ABOi, ABO incompatibility; EGFR, estimated glomerular filtration rate by the Modification of Diet in Renal Disease (MDRD) formula; AST, aspartate aminotransferase; ALT, alanine aminotransferase; WBC, white blood cell count; NLR, neutrophil lymphocyte ratio; CNI, calcineurin inhibitor; MMF, mycophenolate mofetil or mycophenolic acid; EV, everolimus.

**Table 2:** Uni- and multivariate logistic regression model evaluating factors associated with vaccine nonresponsiveness.

Parameters	Univariate			Multivariate		
	OR	95% CI	*p*-Value	OR	95% CI	*p*-Value
Age (years) >65 vs. ≤ 65	0.695	0.397–1.218	0.204			
Type of LT DDLT vs. LDLT	1.268	0.674–2.382	1.268			
Alcohol Yes vs. No	2.018	1.081–3.767	0.027			
ABOi Yes vs. No	5.704	1.774–18.340	0.003	6.053	1.533–23.904	0.010
WBC count (uL) <4000 vs. ≥4000	2.871	1.490–5.533	0.002	2.841	1.350–5.979	0.006
Lymphocyte count (%) <20 vs. ≥20	2.506	1.343–4.675	0.004	2.648	1.162–6.038	0.021
NLR <2.25 vs. ≥2.25	1.828	1.038–3.215	0.037			
Hemodialysis Yes vs. No	2.530	1.046–6.120	0.039			
Tacrolimus trough level >6.5 (ng/mL) Yes vs. No	2.023	1.115–3.672	0.020	2.182	1.056–4.509	0.035
No immunosuppressants stopped (CNI + MMF/EV) Yes vs. No	5.005	2.330–10.749	0.000	5.026	1.837–13.754	0.002
No immunosuppressant stopped (CNI only) Yes vs. No	1.316	0.699–2.475	0.395			
Stopped once (MMF/EV) Yes vs. No	2.790	1.241–6.276	0.013			
Stopped twice (MMF/EV) Yes vs. No	0.258	0.144–0.464	0.000			
Triple immunotherapy Yes vs. No	5.456	1.074–27.709	0.041			

Abbreviations: LT, liver transplantation; DDLT, deceased donor liver transplantation; LDLT, living donor liver transplantation; ABOi, ABO incompatibility; WBC, white blood cell count; NLR, neutrophil lymphocyte ratio; CNI, calcineurin inhibitor; MMF, mycophenolate mofetil or mycophenolic acid; EV, everolimus.

**Table 3:** Patient characteristics according to type of vaccination.

Parameters	MRNA (n = 332)	AZ (n = 22)	KT (n = 20)	*p*-Value
Age (years)	63 (56–68)	58.5 (53.2–68.2)	52.5 (49.2–61.7)	0.003
Gender				0.082
Male	249 (75)	20 (90.9)	18 (90)	
Female	83 (25)	2 (9.1)	2 (10)	
Etiology				
HCC	140 (42.2)	6 (27.3)	7 (35)	0.333
Viral hepatitis	199 (59.9)	10 (45.5)	12 (60)	0.407
HBV	52 (15.7)	4 (18.2)	2 (10)	0.745
HCV	13 (3.5)	12 (3.8)	1 (1.7)	1.000
HBV + HCV	52 (15.7)	13 (59.1)	8 (40)	0.000
Alcohol	18 (5.4)	0	0	0.584
Autoimmune				
Type of LT				0.277
DDLT	79 (23.8)	2 (9.1)	5 (25)	
LDLT	253 (76.2)	20 (90.9)	15 (75)	
Time from LT to vaccination (months)	71 (38.2–110.2)	78.5 (58.2–108)	97 (53.5–137.5)	0.206
Interval between Vaccinations (days)	85.5 (46–98)	88 (79.7–96.2)	38.5 (35–40)	0.000
ABOi	8 (2.4)	4 (18.2)	1 (5)	0.008
EGFR (mL/min/1.73 m^2^)	34.5 (20.1–64.8)	30.6 (5–88.9)	66.5 (29.4–78.7)	0.040
Total bilirubin (mg/dL)	0.7 (0.5–1)	0.7 (0.4–1.3)	0.9 (0.5–1.3)	0.513
AST (U/L)	20 (16–27)	19.5 (16.7–36)	21.5 (16.2–26)	0.963
ALT (U/L)	21 (14–34)	21 (11.7–45)	24 (13–43.7)	0.842
WBC (/uL)	5750 (4600–6900)	5450 (3750–6800)	5750 (4900–6675)	0.614
Lymphocyte (%)	28.2 (22.6–34.5)	25.8 (14.4–29.4)	29.9 (19.3–33.6)	0.235
Tacrolimus trough level (ng/mL)	4.7 (3.6–6.4)	3.6 (2.3–5.2)	5 (2.6–6.7)	0.081
Age >65 (years)	128 (38.6)	6 (27.3)	1 (5)	0.007
Time from LT to vaccination <12 (months)	19 (5.7)	1 (4.5)	0	0.853
History of cancer	145 (43.8)	10 (45.5))	7 (35)	0.728
WBC <4000 (/uL)	46 (13.9)	7 (31.8)	2 (10)	0.082
Lymphocytes <20 (%)	56 (16.9)	7 (31.8)	5 (25)	0.113
NLR <2.25	169 (50.9)	10 (45.5)	11 (55)	0.821
EGFR <30 (mL/min/1.73 m^2^)	151 (45.5)	11 (50)	5 (25)	0.176
Hemodialysis	20 (6)	4 (18.2)	2 (10)	0.054
Tacrolimus trough level >6.5 (ng/mL)	77 (23.2)	4 (18.2)	6 (30)	0.661
No immunosuppressants stopped (CNI + MMF/EV)	26 (7.8)	5 (22.7)	1 (5)	0.059
No immunosuppressant stopped (CNI only)	71 (21.4)	9 (40.9)	4 (20)	0.109
Stopped once (MMF/EV)	22 (6.6)	7 (31.8)	2 (10)	0.002
Stopped twice (MMF/EV)	213 (64.2)	1 (4.5)	13 (65)	0.000
Triple immunotherapy	6 (1.8)	-	-	1.000

Variables are expressed as median (interquartile range) or as number (*n*) and percent (%). Abbreviations: HCC, hepatocellular carcinoma; HBV, hepatitis B virus; HCV, hepatitis C virus; LT, liver transplantation; DDLT, deceased donor liver transplantation; LDLT, living donor liver transplantation; ABOi, ABO incompatibility; EGFR, estimated glomerular filtration rate by the Modification of Diet in Renal Disease (MDRD) formula; AST, aspartate aminotransferase; ALT, alanine aminotransferase; WBC, white blood cell count; NLR, neutrophil lymphocyte ratio; CNI, calcineurin inhibitor; MMF, mycophenolate mofetil or mycophenolic acid; EV, everolimus.

**Table 4:** Baseline characteristics of ABO incompatible patients.

Parameters	ABOi (*n* = 13)	ABOc (*n* = 361)	*p*-Value
Age (years)	56 (48–65)	63 (55–68)	0.096
Gender			1.000
Male	10 (76.9)	277 (74.1)	
Female	3 (23.1)	84 (23.3)	
Time from LT to vaccination (months)	40 (24–81.5)	73 (41–117.5)	0.067
Interval between Vaccinations (days)	45 (37.5–90.5)	85 (45–97)	0.111
Etiology			
HCC	4 (30.8)	149 (41.3)	0.449
Viral hepatitis	9 (69.2)	212 (58.7)	0.449
HBV	1 (7.7)	57 (15.8)	0.701
HCV	0 (0)	13 (3.6)	1.000
HBV + HCV	5 (38.5)	68 (18.8)	0.144
Alcohol	1 (5.6)	17 (4.7)	0.479
Autoimmune			
EGFR (mL/min/1.73 m^2^)	21 (11.6–53.9)	37.1 (20.1–66.3)	0.111
Total bilirubin (mg/dL)	1 (0.7–1.6)	0.7 (0.5–1)	0.027
AST (U/L)	23 (17.5–55)	20 (16–27)	0.135
ALT (U/L)	29 (14–63)	21 (14–34)	0.274
WBC (/uL)	5500 (3650–6700)	5700 (4650–6900)	0.219
Lymphocyte (%)	32 (27.7–37)	27.8 (21.9–34.5)	0.045
Tacrolimus trough level (ng/mL)	6.8 (4.3–8.5)	4.7 (3.5–6.2)	0.031

Variables are expressed as median (interquartile range) or as number (*n*) and percent (%). Abbreviations: LT, liver transplantation, HCC, hepatocellular carcinoma; HBV, hepatitis B virus; HCV, hepatitis C virus; EGFR, estimated glomerular filtration rate by the Modification of Diet in Renal Disease (MDRD) formula; AST, aspartate aminotransferase; ALT, alanine aminotransferase; WBC, white blood cell count.

## Data Availability

The data that support the findings of this study are available on request from the corresponding author.

## Supplementary Materials

The following supporting information can be downloaded at https://www.mdpi.com/article/10.3390/vaccines10111827/s1: Table S1: Comparison of side effects after first and second SARS-CoV-2 vaccination in all patients; Figure S1: Comparison of the humoral responses (SARS-CoV-2 NT50 antibodies) under MMF or EV in patients with immunosuppression adjustment. (A) Adjustment once. (B) Adjustment twice. Data are expressed as median and interquartile range. n.s., not significant (Mann-Whitney U test). Abbreviations: MMF, mycophenolate mofetil; EV, everolimus; Figure S2: Serum aminotransferase levels of before (1) and after (2) vaccinations. (A) Adjustment once. (B) Adjustment twice. Data are expressed as median and interquartile range. n.s., not significant (Mann-Whitney U test). Abbreviations: AST, aspartate aminotransferase; ALT, alanine aminotransferase.
