# Supplementary material for: Determinants of Antibody Response to SARS-CoV-2 Vaccines in Liver Transplant Recipients: The Role of Immunosuppression Reduction
Source: Vaccines (Basel). 2022 Oct 29;10(11):1827. doi: 10.3390/vaccines10111827 (PMC9692368; doi:10.3390/vaccines10111827)
Supplement: Supplementary file 1 [file vaccines-10-01827-s001.zip › Supplementary Table S1.pdf]

Supplementary Table S1

Comparison of side effects after first and second SARS-CoV-2 vaccination in all patients

**Side effects**	**MRNA MRNA (n=332)**	**AZ AZ (n=22)**	**KT KT (n=20)**	***p* value**
**First dose**				
None	165 (49.7)	14 (63.6)	18 (90)	0.001
Local pain	142 (42.8)	6 (27.3)	2 (10)	0.007
Fever	24 (7.2)	2 (9.1)	0 (0)	0.476
Chills	7 (2.1)	1 (4.5)	0 (0)	0.618
Headache	8 (2.4)	1 (4.5)	1 (5)	0.312
Malaise	14 (4.2)	1 (4.5)	0 (0)	1.000
Myalgia	2 (0.6)	1 (4.5)	0 (0)	0.301
Fatigue	32 (9.6)	2 (9.1)	1 (5)	0.915
Palpitation	4 (1.2)	0 (0)	0 (0)	1.000
Diarrhea	3 (0.9)	0 (0)	1 (5)	0.215
Rash/Pruritus	3 (0.9)	0 (0)	0 (0)	1.000
Dyspnea	2 (0.6)	0 (0)	0 (0)	1.000
**Second dose**				
None	166 (50)	13 (59.1)	17 (85)	0.008
Local pain	142 (42.8)	7 (31.8)	3 (15)	0.034
Fever	21 (6.3)	2 (9.1)	0 (0)	0.496
Chills	7 (2.1)	1 (4.5)	0 (0)	0.618
Headache	7 (2.1)	1 (4.5)	1 (5)	0.267
Malaise	15 (4.5)	2 (9.1)	0 (0)	0.439
Myalgia	4 (1.2)	1 (4.5)	0 (0)	0.451
Fatigue	27 (8.1)	2 (9.1)	2 (10)	0.726
Palpitation	5 (1.5)	0 (0)	0 (0)	1.000
Diarrhea	3 (0.9)	0 (0)	1 (5)	0.215
Rash/Pruritus	3 (0.9)	0 (0)	0 (0)	1.000
Dyspnea	1 (0.3)	0 (0)	0 (0)	1.000
