# Supplementary figures and images for: Determinants of Antibody Response to SARS-CoV-2 Vaccines in Liver Transplant Recipients: The Role of Immunosuppression Reduction
Source: Vaccines (Basel). 2022 Oct 29;10(11):1827. doi: 10.3390/vaccines10111827 (PMC9692368; doi:10.3390/vaccines10111827)

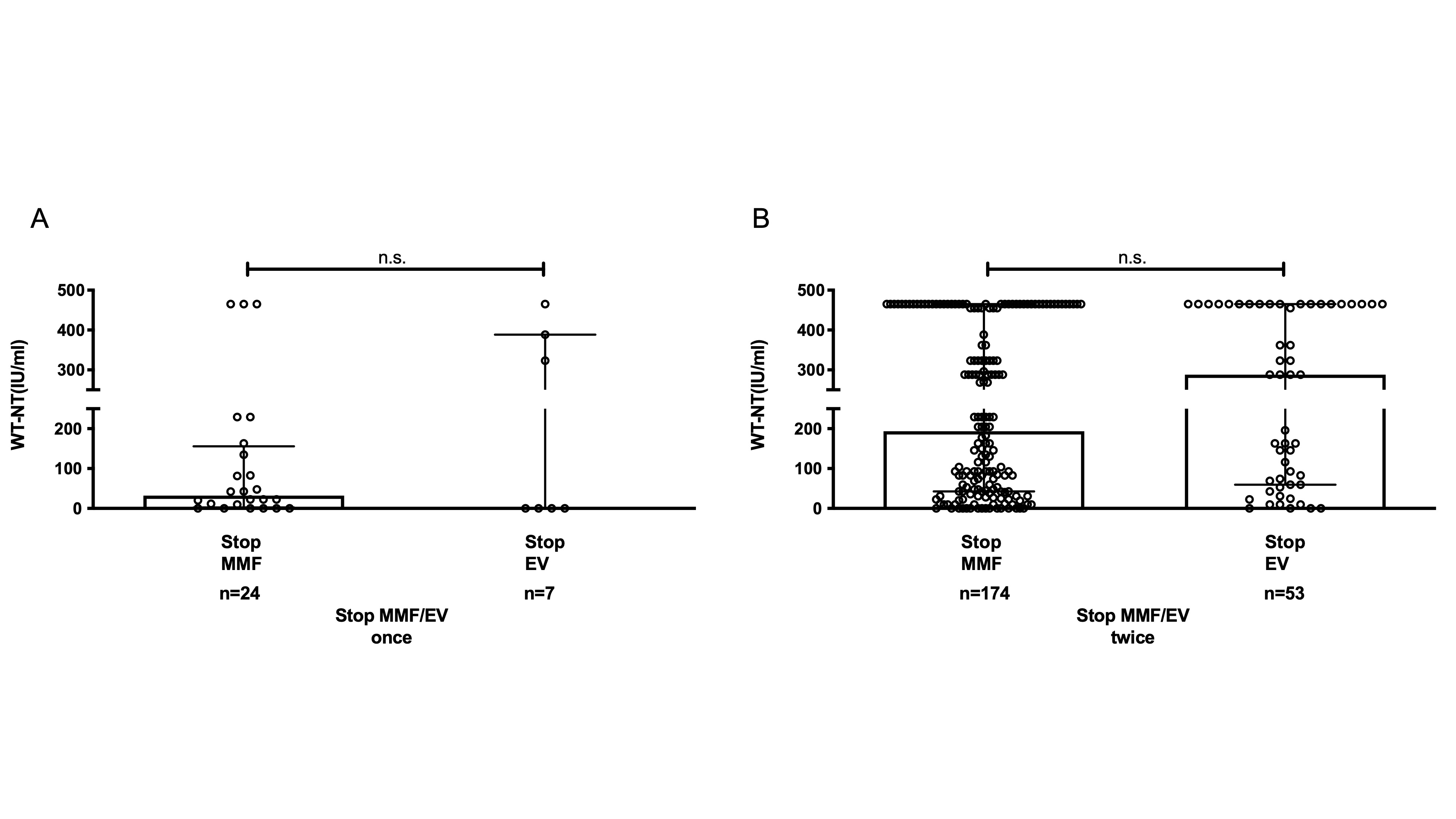

Supplement: Supplementary file 1 [file vaccines-10-01827-s001.zip › Supplementary figure S1.jpg]

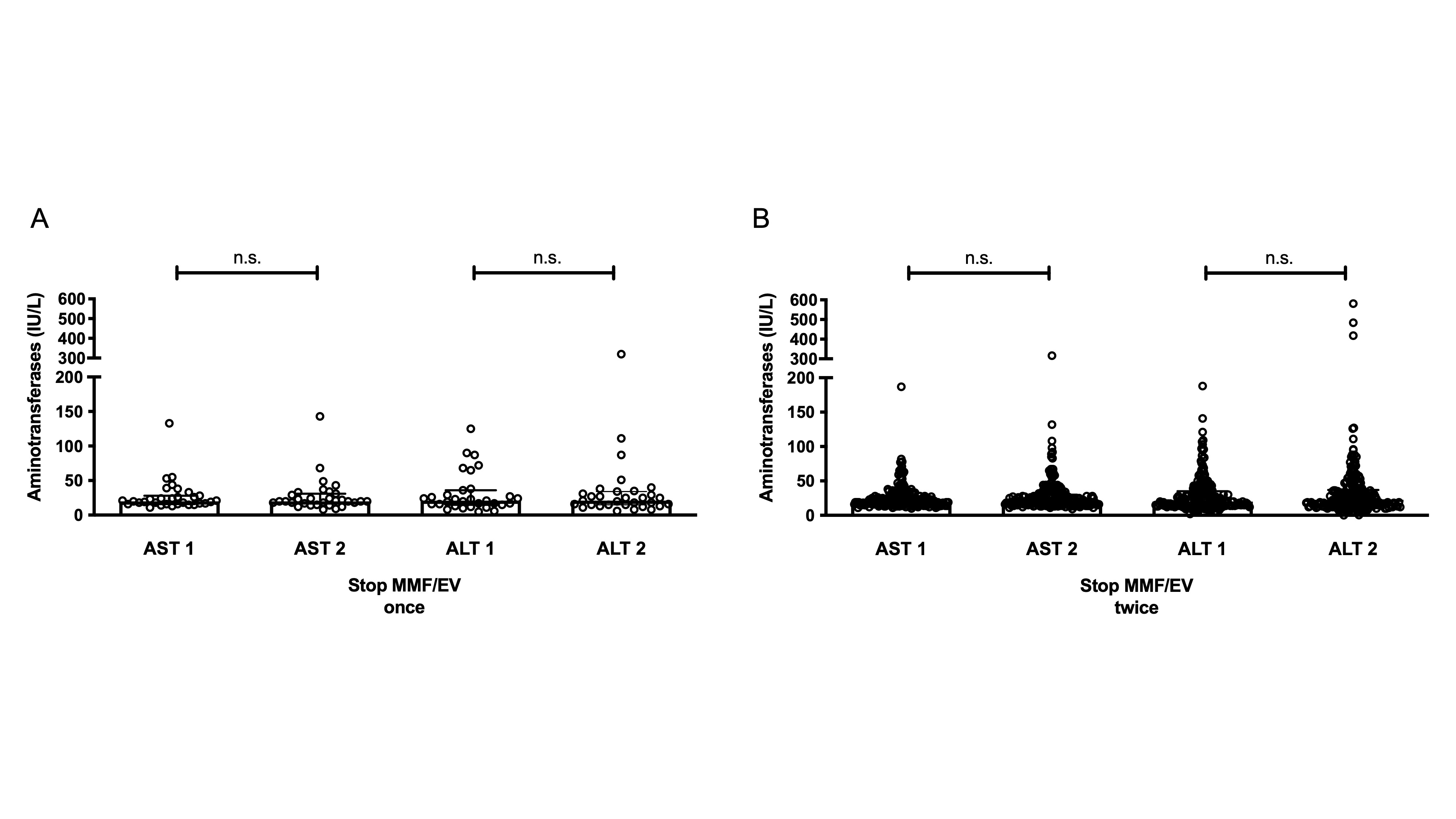

Supplement: Supplementary file 1 [file vaccines-10-01827-s001.zip › Supplementary figure S2.jpg]
